# Different landscape effects on the genetic structure of two broadly distributed woody legumes, *Acacia salicina* and *A. stenophylla* (Fabaceae)

**DOI:** 10.1002/ece3.6952

**Published:** 2020-10-31

**Authors:** Francisco Encinas‐Viso, Christiana McDonald‐Spicer, Nunzio Knerr, Peter H. Thrall, Linda Broadhurst

**Affiliations:** ^1^ Centre for Australian National Biodiversity Research CSIRO Canberra ACT Australia; ^2^ The Australian National University Canberra ACT Australia; ^3^ CSIRO Agriculture & Food Canberra ACT Australia

**Keywords:** Australia, connectivity, gene flow, habitat fragmentation, landscape genetics, population structure, resistance surfaces

## Abstract

Restoring degraded landscapes has primarily focused on re‐establishing native plant communities. However, little is known with respect to the diversity and distribution of most key revegetation species or the environmental and anthropogenic factors that may affect their demography and genetic structure. In this study, we investigated the genetic structure of two widespread Australian legume species (*Acacia salicina* and *Acacia stenophylla*) in the Murray–Darling Basin (MDB), a large agriculturally utilized region in Australia, and assessed the impact of landscape structure on genetic differentiation. We used AFLP genetic data and sampled a total of 28 *A. salicina* and 30 *A. stenophylla* sampling locations across southeastern Australia. We specifically evaluated the importance of four landscape features: forest cover, land cover, water stream cover, and elevation. We found that both species had high genetic diversity (mean percentage of polymorphic loci, 55.1% for *A. salicina* versus. 64.3% for *A. stenophylla)* and differentiation among local sampling locations (*A. salicina*: Φ_PT_ = 0.301, 30%; *A. stenophylla*: Φ_PT_ = 0.235, 23%). Population structure analysis showed that both species had high levels of structure (6 clusters each) and admixture in some sampling locations, particularly *A. stenophylla*. Although both species have a similar geographic range, the drivers of genetic connectivity for each species were very different. Genetic variation in *A. salicina* seems to be mainly driven by geographic distance, while for *A. stenophylla*, land cover appears to be the most important factor. This suggests that for the latter species, gene flow among populations is affected by habitat fragmentation. We conclude that these largely co‐occurring species require different management actions to maintain population connectivity. We recommend active management of *A. stenophylla* in the MDB to improve gene flow in the adversity of increasing disturbances (*e.g.,* droughts) driven by climate change and anthropogenic factors.

## Graphical abstract

The environmental factors driving genetic connectivity of woody legumes in one of Australia's most impacted regions, the Murray Darling Basin (MDB), are poorly understood. Here, we studied the effects of habitat fragmentation and geographical barriers on the gene flow of two broadly distributed Acacia species in the MDB. We found that despite being ecologically similar, each Acacia species had different factors affecting their genetic connectivity in the MDB, with important effects of habitat fragmentation in only one of the species.

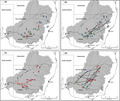

## 1 INTRODUCTION

Environmental changes through space and time can produce genetic differentiation (Fenderson et al., 2020). However, determining the role of specific environmental factors that cause genetic differentiation is still challenging. Changes in the landscape produced by climate change and intensified land use can generate a severe decrease of genetic connectivity and population viability in many plants and animals (Frankham et al., 2010). Therefore, the pervasive effects of global changes, and particularly habitat fragmentation, increase extinction risk of native species in urban and agriculturally intensified areas even in apparently resilient plant species (Vranckx et al., 2012; Young et al., 1996). Shrubby legumes belonging to the genus *Acacia* are highly diverse and widespread across the Australian continent. Acacias form major components of many ecosystems across the continent including many arid ecosystems with poor soils (Bui et al., 2014; Maslin et al., 2003) and play an important role in ecosystem functioning including through the provision of resources and habitat to a broad range of insects and animals (Wandrag et al., 2015; Ward & Branstetter, 2017; Young et al., 2008). It also helps rapid colonization supporting ecosystem recovery following disturbance (Spooner, 2005). Consequently, acacias often play a critical role in the restoration of highly degraded areas (Jeddi & Chaieb, 2012).

Restoration using *Acacia* species primarily occurs within regions where fragmentation of native vegetation is extensive (Doi & Ranamukhaarachchi, 2013; Jeddi & Chaieb, 2012). Fragmentation of large and continuous vegetation results in smaller, more isolated populations, often with lower genetic diversity, an increased risk of further genetic loss through drift and elevated inbreeding (Aguilar et al., 2006, 2008; Hamrick, 2004; Young et al., 1996). Consequently, there are risks associated with using seed crops from small populations for restoration purposes. Our understanding, however, about how landscape fragmentation and other environmental factors (e.g., elevation) shape patterns of gene flow in Australian *Acacia* species remains unclear. A recent meta‐analysis of patterns of genetic diversity highlighted that Australian species generally follow global expectations when factors including range size, form, and abundance are considered (Broadhurst et al., 2017). This study also found that genetic diversity is lower in Australian shrubs (primarily acacias) when compared to trees or herbs and that population genetic structure (F_st_/G_st_) in shrubs and trees was estimated to be twice that observed in global studies. While observations such as these are useful for high‐level comparisons, understanding the major drivers of among‐species variation in genetic diversity and structure may be more important for guiding conservation decisions.

**FIGURE 1:**
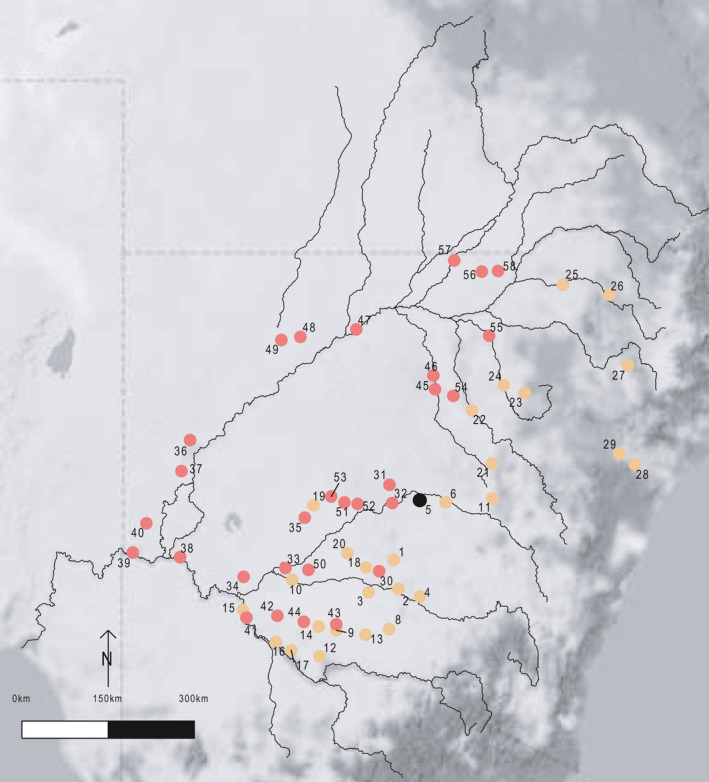
Sampling locations for *A. salicina* (orange filled circles) and *A. stenophylla* (red filled circles). Gray shading indicates extent of Murray Darling Basin. Sampling location *5* (black filled circle) indicates site where both species were collected

Here, we compare genetic diversity, population genetic structure, and landscape genetics using AFLP data in two functionally similar shrubby legumes (*Acacia salicina* and *Acacia stenophylla*) to improve our understanding of the main environmental factors shaping genetic connectivity in these two species. *Acacia salicina* and *Acacia stenophylla* are both broadly distributed across the Murray Darling Basin (MDB) (Figure 1) in eastern Australia, one of Australia's most large river system that has been extensively used for agricultural production (Cai & Cowan, 2008). Importantly, these two species however have partially contrasting life‐history and environmental requirements. *A. salicina* is a perennial woody shrub that mainly occurs in semi‐arid habitats and it is a very successful colonizer of degraded areas with high tolerance of bare soil (Grigg & Mulligan, 1999). This species has been introduced successfully in different parts of the world to revegetate degraded areas and restore soil conditions (Jeddi & Chaieb, 2010), and it is invasive in some arid areas of Israel (Jeddi & Chaieb, 2012). Although not much is known about seed dispersal mechanisms of *A. salicina*, some evidence suggests that birds can disperse their seeds (O’Dowd & Gill, 1986). *A. stenophylla* is a small woody shrub that mainly occurs in riparian ecosystems of Australian river dryland areas. This species provides nesting habitat for many birds in floodplains of inland Australia, and its main seed dispersal mechanism is through hydrochory (Murray et al., 2019). Thus, this species might have specific patterns of genetic structure and diversity modulated by downstream unidirectional gene flow through the MDB river system (Ritland, 1989).

Given their ecological and biological differences and to uncover the effects of landscape fragmentation and other environmental factors on genetic connectivity, we have formulated the following hypotheses: We hypothesize that these two species potentially have different gene flow connectivity patterns through the landscape. We expect *A. stenophylla* to be more sensitive to habitat fragmentation, historical changes in water fluctuations of the MDB (Cai & Cowan, 2008), and being affected by hydrological connectivity directly shaping its genetic structure and diversity, while *A. salicina* seems more resilient and can quickly (re)colonize degraded areas (Grigg & Mulligan, 1999; Jeddi & Chaieb, 2012) and the river system might act as geographic barriers for gene flow. To test these hypotheses, we were interested in determining: (a) Did levels of genetic diversity differ between the two species? (b) Did population genetic structure differ between the two species? And 3. if differences between the two species were evident, could this be explained by environmental factors, such as elevation and/or habitat fragmentation?

## 2 METHODS

### 2.1 Site selection and collection of genetic material

Location data from herbarium specimen records of the Australian Virtual Herbarium were obtained to guide the selection of sites. A survey was then conducted to multiple agricultural areas of the MDB (New South Wales, Australia). We selected sites where the number of mature individuals of *A. stenophylla* and *A. salicina* exceeded 20–30 trees and distance between locations was greater than 30 km (Thrall et al., 2007). A total of 28 *A. salicina* and 30 *A. stenophylla* sampling locations were collected from across the MDB in southeastern Australia (Figure 1). 25 and 28 of those sampling locations of *A. stenophylla* and *A. salicina;* respectively, were located close to riverbanks or water streams. Phyllode material was collected from up to 30 trees in each sampling location, kept cool during transport to the laboratory, lyophilized for 2–5 days (Flexi‐Dry MP FTS Systems, USA), and stored for DNA analysis.

### 2.2 DNA extractions and AFLP genotyping

DNA was extracted from ~10 mg of dried tissue ground to a fine powder using 3‐mm tungsten carbide beads in a Retsch MM300 mixer mill using the Qiagen 96‐well DNEasy Extraction Kit (Qiagen, Melbourne) following the manufacturer's protocol. AFLP amplification largely followed that of (Vos et al., 1995) with the exceptions that 500ng of genomic DNA was digested for each sample using *EcoR*I‐*Mse*I, the *Eco*I‐A*‐Mse*I‐C preamplification reaction was diluted 1:30 prior to selective amplification and selective amplification primers were fluorescently labeled. Initial screening of 12 primer combinations identified six with polymorphic and repeatable banding patterns (Eco‐AGC/Mse‐CTC (FAM), Eco‐AGT/Mse‐CTC (PET), Eco‐ACC/Mse‐CTC (VIC), Eco‐AGC/Mse‐CTG (FAM), Eco‐ACC/Mse‐CTG (VIC), Eco‐AGA/Mse‐CTG (NED)) (dye used shown in brackets). Amplicons were visualized on an ABI 3130XL sequencer using a LIZ 500‐bp internal standard (Applied Biosystems) and scored using GeneMapper Version 4.0 software (Applied Biosystems). A binary matrix of present (1) and absent (0) bands was constructed for each species. Ninety‐five samples from 3 to 4 populations from across the geographic range of both *A. salicina* and *A. stenophylla* were run twice for each of the proposed primer pairs to test for reproducibility across a range of 120 to 450 bp. Markers with an error rate of >5% were discarded with the error rate for markers selected for *A. salicina* ranging from 0–3.4 and for *A. stenophylla* being 1.5–4.3. A negative control was run with every set of samples in a 96‐well block.

### 2.3 Genetic data analyses

The binary data matrix of each species was used to estimate the percentage of polymorphic loci (%P) and expected heterozygosity (*H*_e_) for each sampling location using GenAlEx version 6.41 (Peakall & Smouse, 2006). We also checked for sample size effects for both species (see Figure S12), and we did not find major effects on genetic diversity (*H_e_*); except for sampling location *48* (*N* = 7) of *A. stenophylla*, which showed significant differences of genetic diversity (*H*_e_) (Wilcoxon test: *W* = 322,230, *p* < .05) between all sample sizes. GenAlEx was also used for an analysis of molecular variance (AMOVA (Excoffier et al., 1992)) to determine the distribution of genetic variation among sampling locations for both species with the significance of Φ_PT_ (analogue of *F*_ST_, population differentiation statistics) based on 999 permutations. An exploratory analysis of population genetic structure was undertaken using principal coordinate analyses (PCoA) based on between‐plant pairwise genetic distances (Φ_PT_) using the GenAlEx covariance standardized method with the first two dimensions then plotted. These analyses identified divergent sampling locations in each species, which were removed and the analyses rerun. STRUCTURE version 2.3.2 (Pritchard et al., 2000) was used to determine population structure without prior knowledge of population affinities based on the admixture model, a 50,000 burn‐in followed by 500,000 MCMC repetitions, a uniform prior for alpha, an initial alpha of 1 and allele frequencies correlated among sampling locations. The optimal number of *K*‐clusters was determined with the ad hoc statistic Δ*K* (Evanno et al., 2005) using Structure Harvester v0.6.6 ( Earl & vonHoldt, 2012) from five runs for each *K* = 2–10.

The *popgraph* R package (Dyer, 2009) was used to create population graphs that described the distribution of genetic variation among sampling locations for each species. This graph–theory approach simultaneously identifies genetic covariance structures among subpopulations, does not assume a priori hierarchical or bifurcating statistical models of population arrangement, and is independent of evolutionary assumptions that aim to minimize Hardy–Weinberg and linkage disequilibrium within populations (Dyer & Nason, 2004). Populations are represented as nodes with node diameter representing the level of within‐site heterozygosity, lines connecting nodes show populations that are not significantly genetically differentiated with line length representing among‐site genetic variation (Dyer & Nason, 2004). Paths connecting populations were also examined for “extended edges,” designating long‐distance dispersal, and “compressed edges,” indicating topological or ecological sources of vicariance, both of which were identified by chi‐square tests at α = 0.05.

### 2.4 Resistance surface analysis

To assess the effect of landscape structure on genetic differentiation, we estimated four explanatory variables for each species: (a) “forest cover” based on a continuous forest cover map for 2000 (University of Maryland: http://earthenginepartners.appspot.com/science‐2013‐global‐forest/download.html) where every pixel contained a value of forest cover [0,100], (b) “land cover” based on a categorical Global Land Cover Map for 2009 (GlobCover: http://due.esrin.esa.int/globcover) where every pixel contained a land cover class code, (c) “water stream cover” based on categorical “Surface Hydrology Lines” map of Australia (http://pid.geoscience.gov.au/dataset/ga/83130), and (d) elevation based on a GEODATA 9 Second digital elevation model (DEM) version 3 2008 for the main study area (https://data.gov.au/data/dataset/0fc3357c‐5852‐4e6b‐992c‐c78bd10e9234) where every pixel contained an elevation value expressed in meters. To minimize border effects, we cropped all rasters to the extent of the study region for both *Acacia* species and masked them using a shape file of Australia. We also standardized by reprojecting (EPSG:4326) and aggregating all rasters to similar size grid cells. Spatial data were prepared using the R packages *raster* (Hijmans & van Etten, 2012) and *rgdal* (Bivand, Keitt, & Rowlingson, 2020). The “forest cover” was aggregated by a factor of 3, using the mean function, “land cover” was similarly aggregate by a factor of 30 and reclassified as outlined below. The DEM was rescaled (min 0.001 – max 1) and also aggregated by a factor of 3 using the mean function.

We used circuit theory (McRae et al., 2008) to estimate the resistance to gene flow between sampling locations of both species for each of the explanatory variables (forest cover, land cover, water stream cover, elevation) using Circuitscape v4.0 (McRae, 2006) to estimate pairwise resistance distances. Because we hypothesized higher gene flow across intact forest remnants than between regions predominantly covered by agricultural areas, we created resistance surfaces where agricultural area pixels had higher resistance values. We created two separate resistance surfaces: one using land cover and one using forest cover maps. We used raw values of forest cover rasters (Figure S5) and transformed the categorical values of land cover rasters to numerical resistance values ranging between 0 and 1 (Figure S4). More specifically, we assigned a minimal resistance of 0.1 to all forested land cover classes, medium resistance values (0.4–0.5) to areas containing fragmented habitats, and a maximal resistance of 0.9 to all other classes (agricultural and permanent snow areas). We also tested two more the hypotheses: (a) that elevation influenced genetic connectivity with mountain ranges being a potential barrier to *Acacia* gene flow and (b) that water streams might be positively affecting gene flow between Acacia populations (particularly *A. stenophylla*). To do this, we created resistance surfaces with pixels at higher elevations having higher resistance values using the raw elevations from the DEMs as resistance values for each pixel (Figure S6) and we created a “water stream cover” conductance surfaces only taking major perennial watercourses with conductance values of 1 (Figure S7). Finally, to test for isolation by geographic distance (IBD), we created null‐model rasters by replacing all values of the forest cover rasters with 0.5 and calculated resistance distances between sampling locations. Because Circuitscape does not accept zero resistance values, we replaced zero values in all rasters with 0.0001.

### 2.5 Landscape genetic analysis

We used conditional genetic distance (Dyer & Nason, 2004) as our response variable and it was calculated using the R package *gstudio* (Dyer, 2009). This is an interindividual genetic distance, which considers genetic covariation among all studied sampling locations. As explanatory variables, we used the various distance matrices described above: geographic distance, forest cover, land cover, water stream cover, and elevation. We tested correlations among genetic and environmental distances according to the different scenarios considered using: (a) Mantel (Mantel, 1967) and partial Mantel tests (i.e., to control for spatial autocorrelation using the geographic distance matrix); and (b) multiple regression on distance matrices (MRM, (Lichstein, 2007)). Mantel tests were applied using the R package *vegan* (vs. 2‐0‐10) (Oksanen et al., 2017) and estimated significance based on 10 000 permutations. MRM were implemented in the R package *ecodist* vs. 1.2‐9 (Goslee & Urban, 2007) by building an initial model. For each covariate, we then estimated regression coefficients and associated *p*‐values based on 10,000 permutations. To select the model that best explained genetic differentiation, we used a backward elimination model selection approach as described in Legendre and Legendre (2012). The first model included all variables: geographic distance, forest cover, land cover, water stream cover, and elevation. The variable showing the highest nonsignificant p‐value was removed and we repeated this procedure until all variables included in the analysis showed p‐values lower than 0.05. We corrected p‐values obtained for Mantel tests and MRM models for multiple testing using the Benjamini and Hochberg (1995) method as implemented in the *stats* package in R.

## 3 RESULTS

### 3.1 Genetic diversity and population genetic structure

Genetic diversity measures varied among sampling locations within species as well as between *A. salicina* and *A. stenophylla*. The percentage of polymorphic loci ranged from 36%–76.4% in *A. salicina* and 46.2%–83.9% in *A. stenophylla* while expected heterozygosity for *A. salicina* was 0.117–0.216 and 0.151–0.280 in *A. stenophylla* (Table 1). Although differences were also evident at the species level for mean percentage of polymorphic loci (55.1% for *A. salicina* vs. 64.3% for *A. stenophylla*) and mean expected heterozygosity (0.162 for *A. salicina* vs. 0.214 for *A. stenophylla*), slight differences in the primer pair combinations used to generate the datasets suggest caution when comparing these results. The AMOVAs indicated substantial and significant differences in genetic variation among sampling locations of both species (*A. salicina*: Φ_PT_ = 0.301, 30.1%, *p* = .010; *A. stenophylla*: Φ_PT_ = 0.235, 23.5%, *p* = .010). Significant isolation by distance was also detected in both species (*p* < .001).

**TABLE 1:** Genetic diversity measures for *A. salicina* and *A. stenophylla*. *N*, number of plants; %P, percentage of polymorphic loci; *H*_e_, expected heterozygosity. Bold values show mean % P and *H_e_* values per species

*A. salicina*				*A. stenophylla*			
Site No.	*N*	%P	*H*_e_ (*SE*)	Site No.	*N*	%P	*H*_e_ (*SE*)
1	10	40.0%	0.117 (0.010)	5	12	51.1%	0.175 (0.013)
2	8	45.5%	0.151 (0.012)	30	10	52.0%	0.195 (0.014)
3	18	49.5%	0.140 (0.011)	31	21	68.2%	0.228 (0.013)
4	23	42.9%	0.121 (0.010)	32	22	47.5%	0.166 (0.013)
5	17	52.0%	0.153 (0.011)	33	24	59.6%	0.190 (0.013)
6	14	42.2%	0.120 (0.011)	34	30	60.5%	0.188 (0.013)
8	21	65.8%	0.192 (0.011)	35	20	47.1%	0.151 (0.013)
9	28	53.5%	0.150 (0.011)	36	25	73.1%	0.245 (0.013)
10	29	55.6%	0.154 (0.011)	37	20	71.3%	0.243 (0.013)
11	15	36.0%	0.118 (0.011)	38	21	73.5%	0.248 (0.013)
12	10	37.8%	0.126 (0.011)	39	29	64.6%	0.211 (0.013)
13	18	67.3%	0.216 (0.012)	40	26	61.0%	0.190 (0.013)
14	18	52.0%	0.166 (0.012)	41	24	68.2%	0.233 (0.013)
15	30	68.0%	0.211 (0.012)	42	24	83.9%	0.275 (0.013)
16	17	62.9%	0.210 (0.012)	43	24	73.5%	0.241 (0.013)
17	23	76.4%	0.203 (0.011)	44	16	54.3%	0.198 (0.014)
18	19	62.5%	0.174 (0.011)	45	18	67.7%	0.230 (0.013)
19	21	56.0%	0.159 (0.011)	46	21	78.0%	0.280 (0.013)
20	21	52.7%	0.156 (0.011)	47	24	71.3%	0.215 (0.013)
21	26	42.5%	0.129 (0.011)	48	7	46.2%	0.168 (0.014)
22	25	62.5%	0.185 (0.012)	49	26	70.9%	0.224 (0.013)
23	28	60.4%	0.163 (0.011)	50	22	66.4%	0.215 (0.013)
24	19	57.8%	0.169 (0.011)	51	27	65.5%	0.217 (0.013)
25	24	66.9%	0.194 (0.011)	52	19	67.7%	0.232 (0.014)
26	26	56.7%	0.161 (0.011)	53	29	71.3%	0.216 (0.012)
27	19	49.8%	0.152 (0.011)	54	22	69.1%	0.233 (0.014)
28	28	67.3%	0.194 (0.011)	55	19	59.2%	0.202 (0.014)
29	30	59.6%	0.155 (0.011)	56	24	63.7%	0.217 (0.013)
Mean		**55.1%**	**0.162 (0.002)**	57	29	65.5%	0.208 (0.013)
				58	26	56.1%	0.181 (0.013)
				Mean		**64.3%**	**0.214 (0.001)**

The first two principal coordinates axes accounted for 56.9% of the total variation in *A. salicina* and 49.3% of the variation for *A. stenophylla* (Figure S1). Divergent sampling locations were evident for both of these PCos, namely, *A. salicina* plants from sampling locations 13–16 were located in negative PCo1 space (Figure S1a) and a group of *A. stenophylla* plants from sampling locations 37, 38, 42, 44, and 46 were in negative PCo1 and PCo2 space (Figure S1c).

**FIGURE 2:**
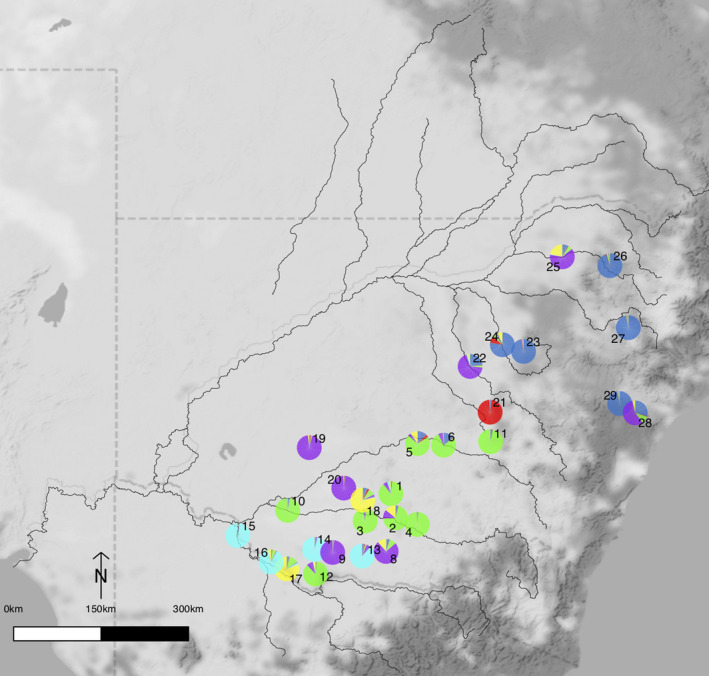
STRUCTURE output of *A. salicina* for *K = 6*. Geographical location of *A. salicina* sampling locations colored to match *K* = 6. Sampling location numbers as per Table 1. Colors distinguish the genetic clusters inferred from STRUCTURE

**FIGURE 3:**
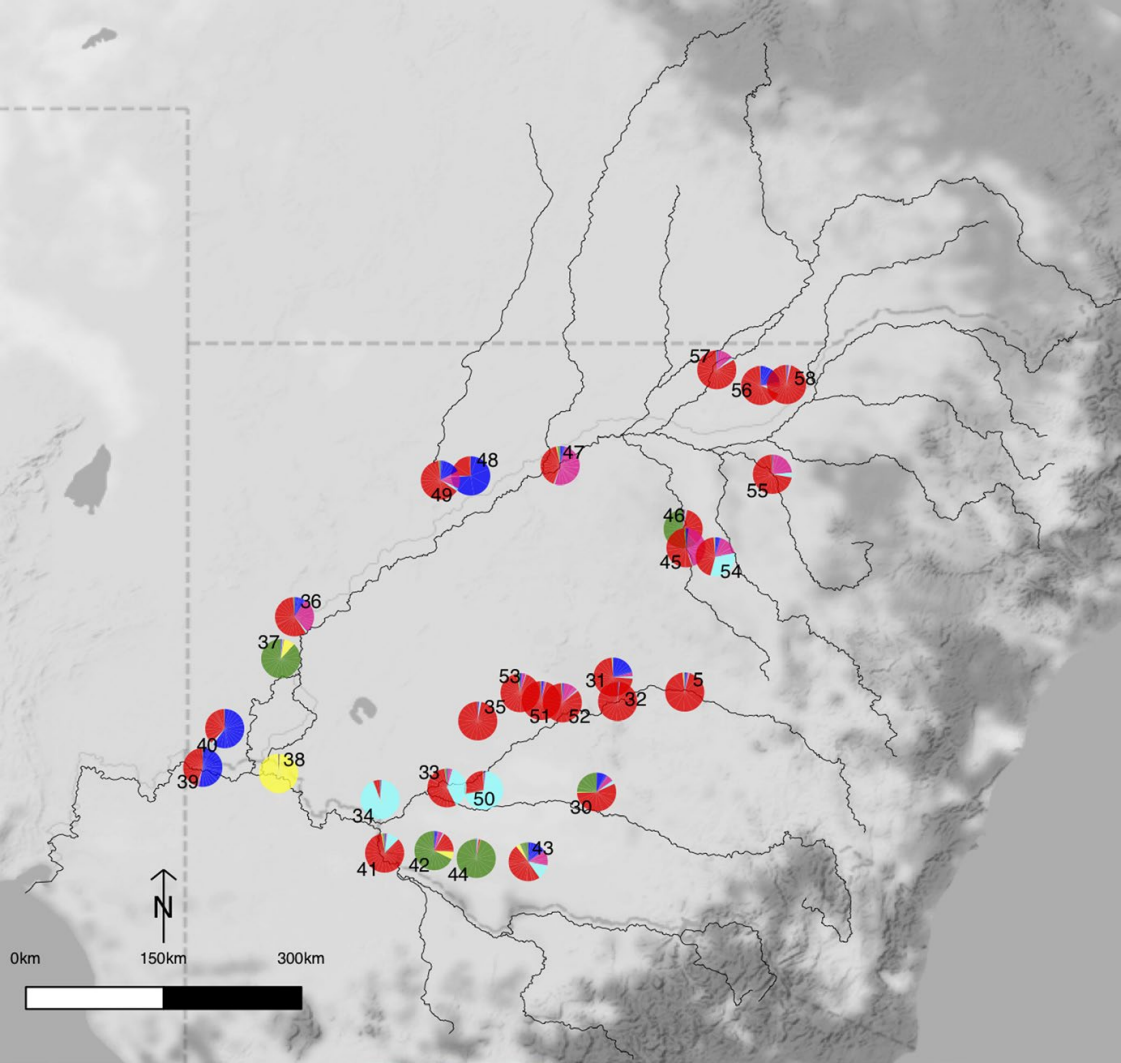
STRUCTURE output of *A. stenophylla* for *K = 6*. Geographical location of *A. stenophylla* sampling locations colored to match *K* = 6. Sampling location numbers as per Table 1. Colors distinguish the genetic clusters inferred from STRUCTURE

The analyses done by STRUCTURE showed that most likely there are six genetic clusters (*K* = 6) for both species (Figures 2 and 3) based on the statistic ∆*K,* although there was some evidence that a smaller number of clusters (i.e., 3) might also be present (Figure S11a,b). There was little evidence of admixture in many of the *A. salicina* plants (Figure 2 and Figure S2). For example, some northern sampling locations (23, 24, 26, 27, and 29) were strongly associated with cluster 1 (dark blue); however, some plants in sampling location 24 had associations with cluster 4 (yellow) and 6 (red). In contrast, other *A. salicina* sampling locations (e.g., 2, 5, 6, 22, 25, and 28) showed evidence of admixture or potentially immigration from sampling locations belonging to other groups. The *A. salicina* clusters were somewhat geographically partitioned across the study region (Figure 2 and Figure S9) with cluster 1 (dark blue) found to the northeast, cluster 2 (light blue) restricted to the most southerly edge while cluster 3 (purple) was broadly distributed. Clusters 4 (yellow) and 5 (green) were distributed broadly in the southern half while the single cluster 6 (red) sampling location is found to the northeast. Two sampling locations (22 and 28) were a mixture of several clusters and could not be assigned to a single cluster at >70%. Results from *K = 3*, the second highest ∆*K*, show a clear geographic pattern of northern and southern clusters (Figure S11a). Unlike *A. salicina*, many *A. stenophylla* sampling locations were dominated by cluster 1 (red, Figure 3d) and many sampling locations showed evidence of admixture or immigration (Figure 3 and Figure S3). Many sampling locations could not also be assigned to a single cluster at >70%. These results are also clearly observed for *K = 3* (Figure S11b). Geographically the majority of sampling locations assigned to cluster 1 were located in headwaters with sampling locations not easily assigned or those belonging to other clusters located downstream.

**FIGURE 4:**
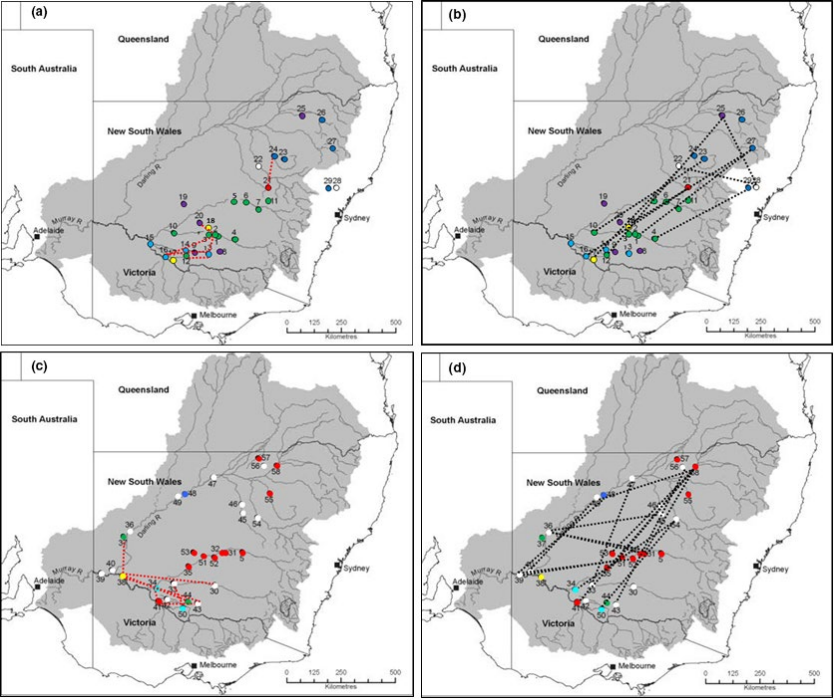
Visualization of *A. salicina* Popgraph compressed (A) (red dashed lines) and extended (B) (black dashed lines) edges for and *A. stenophylla* compressed (C) (red dashed lines) and extended (D) (black dashed lines) edges. Geographical location of *A. stenophylla* and *A. salicina* sampling locations colored to match *K* = 6. White nodes indicate < 70% assignment to a single cluster

Popgraph highlighted a complex web of connected sampling locations (Figure 4, Figures S9 and S10). Few compressed edges indicating ecological or topographical barriers to dispersal were evident in *A. salicina* except among several sampling locations at the southern edge of the study area and between sampling locations 21 and 24 (Figure 4a). Extended edges indicative of long‐distance dispersal linked many of the *A. salicina* sampling locations across broad spatial scales up to 500 km (Figure 4b). More compressed edges were observed in *A. stenophylla*, again at the southern end of the study area (Figure 4c) while extended edges were found across the whole of the region (Figure 4d).

### 3.2 Landscape genetics analysis: Mantel tests and multiple regression on distance matrices (MRM)

We used resistance surfaces of each environmental variable calculated by *Circuitscape* for our landscape genetic analysis on each species. We did not find any significant relationship between genetic clusters and the different resistance surfaces (LC, FC, and DEM) for *A. stenophylla* and *A. salicina*, except for forest cover effects on genetic clusters of *A. salicina* (Kruskal–Wallis χ^2^ = 12.657, *p* = .048). More specifically, the admixed cluster (white) consisting of two sampling locations was mostly related to areas of high forest cover resistance (Figure S7a).

Mantel tests showed that the genetic structure of *A. salicina* was affected by both geographic distance and land cover resistance (*p* < .05), while for *A. stenophylla,* only land cover resistance appeared to be important (Table 2). After correcting by spatial autocorrelation (partial Mantel tests), none of these factors had a significant effect on either of the *Acacia* species (Table 2). However, the MRM model considering only geographic distance was the best model explaining genetic variation between sampling locations in *Acacia salicina* whereas the MRM model including only land cover and elevation was the best model explaining sampling location genetic differentiation for *Acacia stenophylla* (Table 3, Table S1).

**TABLE 2:** Results of Mantel and Partial Mantel correlation tests for *A. salicina* and *A. stenophylla*. For each test, *r*_M_ is provided. Significant results (*p*‐value < .05) after *p*‐value correction are shown in bold and italicized. The variables considered are geographic distance (GD), elevation (DEM), forest cover (FC), water stream cover (WC), and land cover resistance (LC)

	Species	
	*A. salicina*	*A. stenophylla*
Mantel tests		
GD	***0.175***	0.035
DEM	−0.212	−0.217
FC	0.077	−0.093
LC	***0.205***	***0.152***
WC	−0.069	0.041
Partial Mantel tests		
DEM	−0.402	−0.26
FC	0.068	−0.158
LC	0.171	0.179
WC	−0.062	0.043

**TABLE 3:** Best MRM models obtained with backward elimination for both *A. salicina* and *A. stenophylla*. The variables considered are geographic distance (GD), elevation (DEM), forest cover resistance (FC), water stream cover (WC), and land cover resistance (LC). Bold text shows significant values (*p*‐value < .05)

Species	*R* ^2^	*F*	GD	DEM	FC	LC	WC
*A. salicina*	0.03	12.01	**2.38e−05** (*p* = .004)	–	–	‐–	–
*A. stenophylla*	0.117	28.85	–	**275.996** (*p* = .019)	–	**39.81** (*p* = .013)	–

## 4 DISCUSSION

The MDB is Australia's longest river system (area of ~1.0 million square kilometers) and a vital economic resource for the agricultural industry (Cai & Cowan, 2008); however, it is also considered one of Australia's most impacted ecosystems (Cole et al., 2016). Disturbed landscapes, such as the MDB, alter spatial structure and affect plant demography by decreasing population size and increasing population isolation due to geographical distance or barriers in the landscape (Kwak et al., 2009). While habitat fragmentation is expected to diminish gene flow between local populations, ultimately affecting patterns of genetic differentiation and viability (Ellstrand, 1992; Kwak et al., 2009; Young et al., 1996), some evidence suggests that a lack of intervening vegetation may actually increase gene flow (Sork & Smouse, 2006). Our results show that *A. salicina and A. stenophylla* species had distinct patterns of genetic differentiation among populations and that similar elements of landscape structure had an important influence on the observed genetic variation.

Both STRUCTURE and genetic diversity analyses show that *A. salicina* had high genetic differentiation with six distinct genetic clusters. Population graph analysis and PCoA further confirmed these results, showing the effect on genetic differentiation by geographic barriers that separate northern and southern sampling locations. However, STRUCTURE analysis might be overestimating genetic structure of *A. salicina* due to high levels of IBD (Frantz et al., 2009). In contrast, *A. stenophylla* showed high levels of admixture in many sampling locations dominated by a single cluster (1, red) from six genetic clusters detected by STRUCTURE. This suggests that gene flow of *A. stenophylla* is relatively high across many northern and southern sampling locations within its geographical range. However, few sampling locations were composed of distinct genetic clusters [e.g., cluster 3 (green) only present in the south and cluster 4 (black) in the far southwest (see Figure 3). Our findings thus reveal that, contrary to *A. salicina*, *A. stenophylla* is able to maintain higher gene flow across large distances (at least 300 km). Although we did not find an effect of hydrological connectivity, this partially confirms results from a previous study of population genetics of *A. stenophylla,* which shows high levels of population connectivity across the northwest region of the MDB mainly driven by hydrochory (Murray et al., 2019). Seed dispersal through water streams can occur over large distances in this species (>100 km) (Murray et al., 2019). The high levels of genetic connectivity in *A. stenophylla* also provide some evidence showing that gene flow could be maintained in trees despite the landscape fragmentation caused by agricultural production (Byrne et al., 2007).

Our spatial analysis suggests that distinct environmental factors influence genetic differentiation in the two studied species. Geographic distance is the main factor determining *A. salicina* genetic structure, which confirms the findings by STRUCTURE and the visualization of the population graph. Interestingly, this means that habitat fragmentation measured by land cover and forest cover in this area does not seem to have a strong effect on gene flow. Geographical barriers, such as presence of major rivers (Darling and Murray rivers) and geographical distance, seem to shape the observed pattern of genetic variation. However, model fitting (*R* ^2^ = 0.03) was poor, suggesting that other environmental factors, not considered here, might have an effect on the genetic connectivity of *A. salicina*. For example, extensive droughts in the Murray Darling Basin area are known to have an impact on *Acacia* demography (Godfree et al., 2019). However, population graph analysis indicates that gene flow between populations is relatively high based on the number of compressed and extended edges across *A. salicina's* range. Thus, the results overall suggest that gene flow is not affected by habitat fragmentation and it is likely that this species, which is a successful colonizer of disturbed areas (Grigg & Mulligan, 1999), is fairly resilient to fragmentation because it has large seed banks, high growth rate and tolerance to bare soil (Jeddi & Chaieb, 2012).

In the case of *A. stenophylla*, habitat fragmentation (predicted by land cover) did have a significant effect on genetic structure. Thus, genetic connectivity among *A. stenophylla* sampling locations does not seem to be mainly driven by geographic distance (contrary to *A. salicina*) and MRM showed that elevation might also act as a potential barrier for gene flow between sampling locations. Interestingly, given the high levels of admixture in many sampling locations of *A. stenophylla*, the effects of land cover and elevation are likely to explain the presence of distinct genetic clusters in southern sampling locations (green, yellow, and light blue clusters; see Figure 4c,d) where there is high habitat fragmentation produced by extensive areas of agricultural land (Figure S4). Despite these factors, genetic structure and diversity analysis shows that this species is largely unconstrained across its range with long‐distance seed movement possibly helping to maintain homogeneity among sampling locations, especially within rivers and tributaries as it has been shown in a previous study (Murray et al., 2019). Although we did not find a significant effect of water stream cover, occasional one‐directional long‐distance seed dispersal may partly explain high levels of admixture in many sampling locations located at the river margins (Figures 1 and 3).

Although high levels of gene flow have been found in the northern MDB (Upper Murray River) in *A. stenophylla* (Murray et al., 2019), we found that sampling locations in the southern MDB (Lower Murray River) are more structured with lower levels of gene flow (Figures 3 and 4). Evidence from a freshwater fauna study suggests that population divergence and differentiation between Upper and Lower Murray River are recent (~125 years) and likely induced by anthropogenic disturbance (Cole et al., 2016). Water streams of the MDB have been heavily managed for agricultural purposes since European settlement and, in recent times, are known to suffer from lower and more even flow volumes (Adamson et al., 2009; Cai & Cowan, 2008). This suggests that the population connectivity of *A. stenophylla* may now be partially affected by severe water flow fluctuations and management, particular in the Lower Murray River, of the MDB (Oliver & Merrick, 2006).

Several studies have pointed out the negative impacts of habitat fragmentation on plant population viability and genetic diversity (Millar et al., 2014; Young et al., 1996). Our results suggest that a decrease of forested areas can significantly alter genetic differentiation for *A. stenophylla*, but our results do not support that for *A. salicina*. Management actions to improve connectivity of these species (including through water management) need to be tailored accordingly based on our findings. For example, southern sampling locations of *A. stenophylla* who continue to suffer severe water fluctuations that will decrease seed dispersal between populations might be benefited by translocation of individuals where we can target populations with relatively large effective sizes and that are relatively well connected by gene flow to other large populations.

This study provides an important contribution to understanding patterns of genetic differentiation for key plant restoration species in Australia across an important agro‐ecosystem region. Our results show that (a) both species had relatively high levels of genetic diversity and differentiation; (b) both species also had high levels of genetic structure across the MDB, although *A. stenophylla* also showed high admixture levels in several sampling locations; and (c) habitat fragmentation and elevation do not equally affect the genetic connectivity of these two woody legumes supporting our hypothesis. While it seems that *A. salicina* genetic differentiation and connectivity are mainly driven by geographic distance, anthropogenic disturbances in the MDB do have an important impact on gene flow in *A. stenophylla* and it is likely that it affects other less resilient plant species in the region (for example, wetland specialists (Colloff et al., 2014)). Previous studies show that severe impact it is already occurring in freshwater fauna in the MDB (Chessman, 2011; Cole et al., 2016) augmented by the increasing effects of climate change (Adamson et al., 2009; Balcombe et al., 2011). We also suggest that this work could serve as a reference for studies aiming to assess the importance of their associated legume symbionts (nitrogen‐fixing rhizobial bacteria) (Thrall et al., 2005, 2007) to understand how their composition and genetic variation across large geographic scales might be associated with the survival and reproduction of *Acacia* species.

## CONFLICT OF INTEREST

None declared.

## AUTHOR CONTRIBUTION

**Francisco Encinas‐Viso:** Conceptualization (lead); Formal analysis (lead); Investigation (lead); Methodology (lead); Project administration (equal); Software (lead); Supervision (lead); Validation (equal); Visualization (equal); Writing‐original draft (lead); Writing‐review & editing (lead). **Christiana McDonald‐Spicer:** Data curation (equal); Formal analysis (equal); Investigation (equal); Methodology (equal); Visualization (equal); Writing‐review & editing (equal). **Nunzio Knerr:** Data curation (equal); Formal analysis (equal); Investigation (equal); Software (equal); Visualization (lead); Writing‐review & editing (equal). **Peter Thrall:** Conceptualization (equal); Data curation (equal); Funding acquisition (lead); Investigation (equal); Methodology (equal); Project administration (equal); Resources (lead); Supervision (equal); Validation (equal); Writing‐review & editing (equal). **Linda Broadhurst:** Conceptualization (lead); Data curation (equal); Formal analysis (supporting); Funding acquisition (lead); Investigation (equal); Methodology (equal); Project administration (lead); Resources (lead); Software (supporting); Supervision (equal); Validation (equal); Visualization (supporting); Writing‐original draft (supporting); Writing‐review & editing (equal).

## Supporting information

Supplementary Material

## Data Availability

Genetic data of both *Acacia* species will be deposited at CSIRO Data Access Portal.
