# Supplementary material for: Different landscape effects on the genetic structure of two broadly distributed woody legumes, Acacia salicina and A. stenophylla (Fabaceae)
Source: Ecol Evol. 2020 Oct 31;10(23):13476–87. doi: 10.1002/ece3.6952 (PMC7713966; doi:10.1002/ece3.6952)
Supplement: Supplementary file 1 — Supplementary Material [file ECE3-10-13476-s001.docx]

**Supporting Information for:**

”Different landscape effects on the genetic structure of two broadly distributed woody legumes, Acacia salicina and A. stenophylla Fabaceae)”

Francisco Encinas-Viso^1*^, Christiana McDonald-Spicer^1,3^, Nunzio Knerr^1^, Pete H. Thrall^2^ and Linda Broadhurst^1^

**^1^** Centre for Australian National Biodiversity Research, CSIRO, Canberra, ACT, Australia

^2^ CSIRO Agriculture & Food, Canberra, ACT, Australia

^3^ The Australian National University, Canberra, ACT, Australia

*corresponding author email: [francisco.encinas-viso@csiro.au](mailto:francisco.encinas-viso@csiro.au)

**Figure S1.** First two principal co-ordinates (PCo) for all *A. salicina* plants (A) and for all *A. stenophylla* plants (B).

**Figure S2.** Typical bar plot showing STRUCTURE output for individual *A. salicina* plants for *K*=6.

**Figure S3.** Typical bar plot showing STRUCTURE output for individual *A. stenophylla* plants for *K*=6.


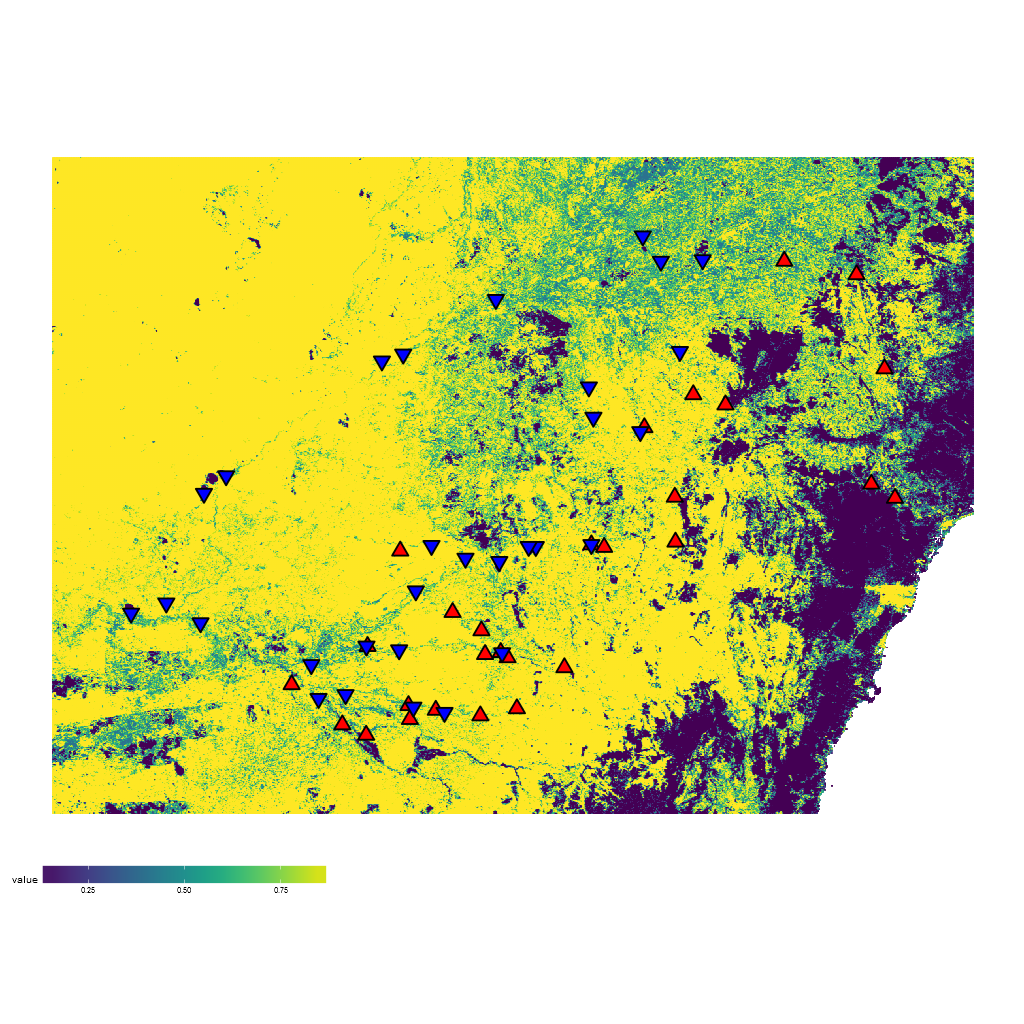


Figure S4. Land cover map of the Murray-Darling Basin. Colours represent transformed values of land cover from low (dark blue) to high (yellow) resistance. Sampling locations are shown for *A. salicina* (red triangles) and *A. stenophylla* (blue triangles).


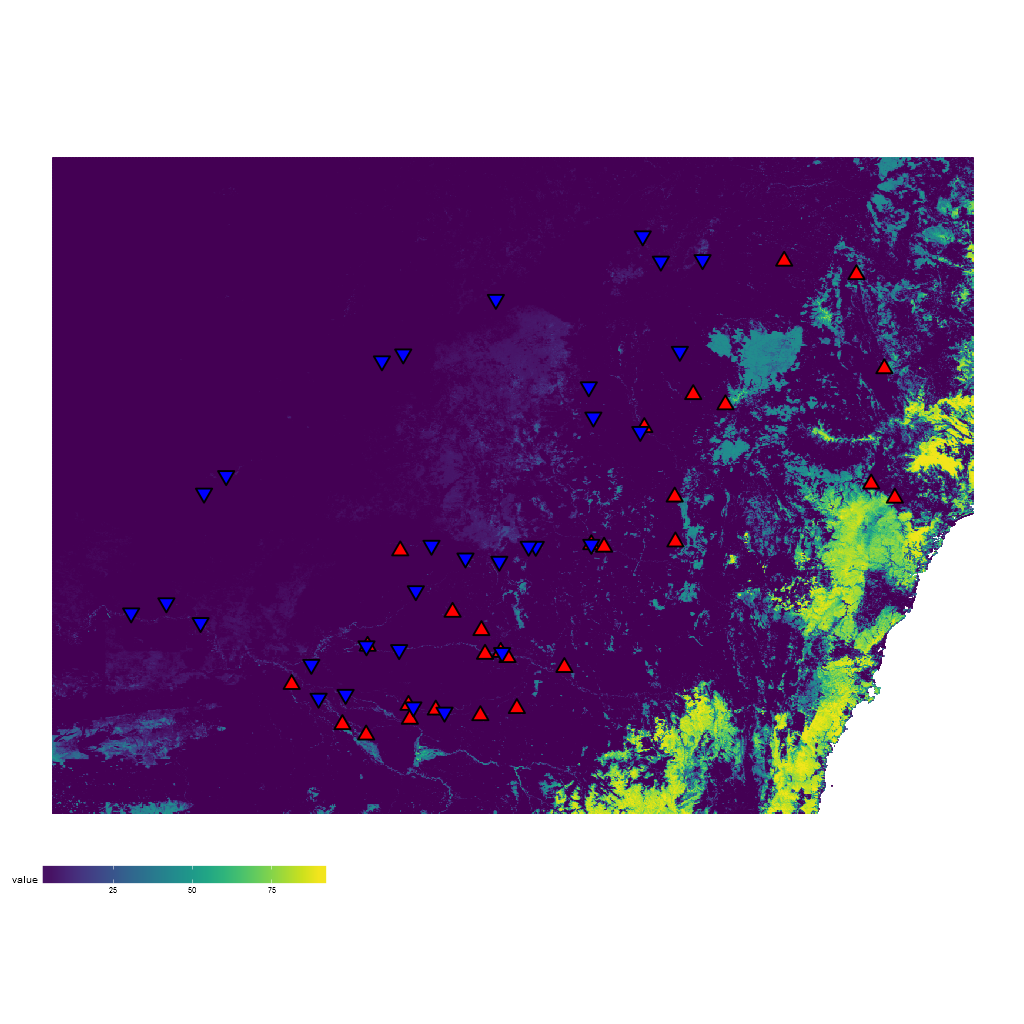


Figure S5. Forest cover map of the Murray-Darling Basin. Colours represent values of forest cover from low (dark blue) to high (yellow) resistance. Sampling locations are shown for *A. salicina* (red triangles) and *A. stenophylla* (blue triangles).


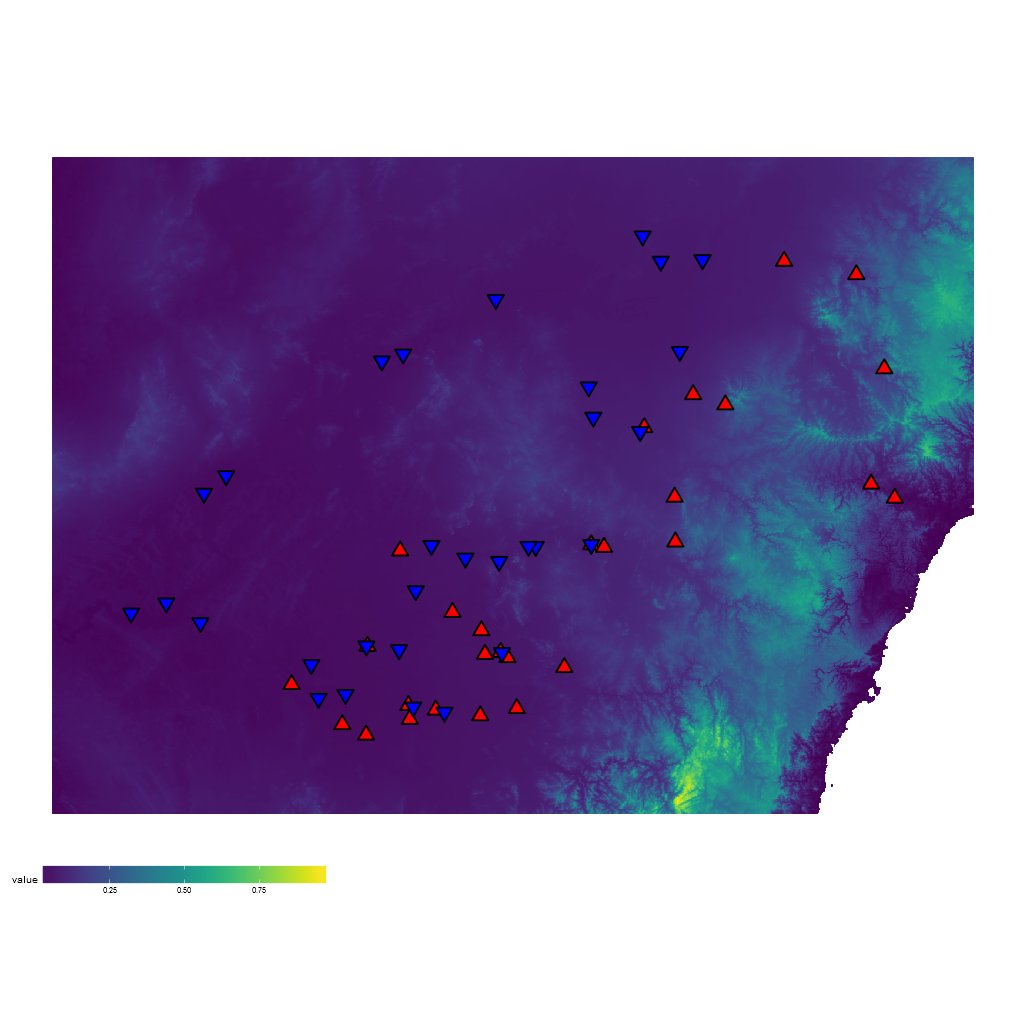


Figure S6. Digital elevation map of the Murray-Darling Basin. Colours represent values of elevation from low (dark blue) to high (yellow) resistance. Sampling locations are shown for *A. salicina* (red triangles) and *A. stenophylla* (blue triangles).


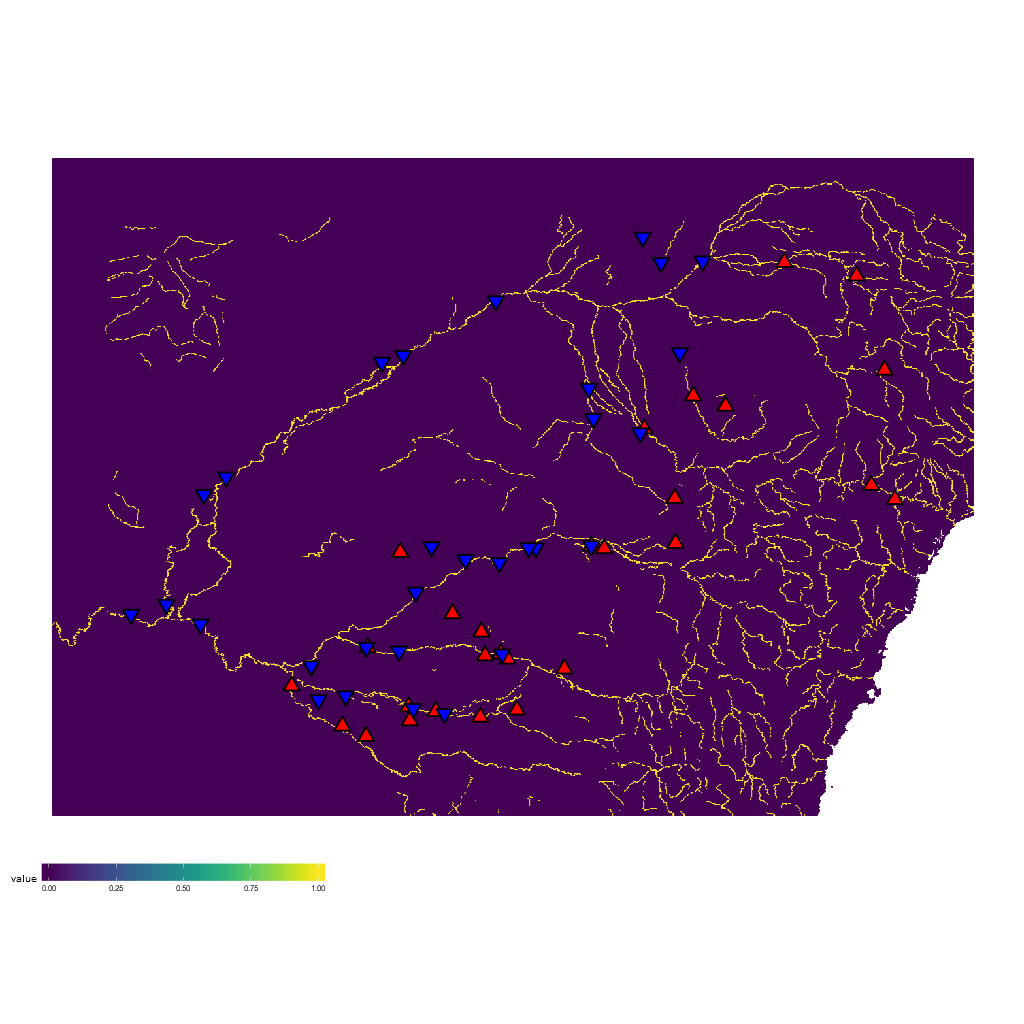


Figure S7. Water stream cover map of the Murray-Darling Basin. Yellow lines represent water streams values of high conductance. Sampling locations are shown for *A. salicina* (red triangles) and *A. stenophylla* (blue triangles).

Figure S8. Variation of resistance values between genetic clusters for *A. salicina* and *A. stenophylla.* Clusters shown for each species were inferred by STRUCTURE analysis (see Results section) and they are shown based on colours represented in Figure 2 and 3 in the main text.


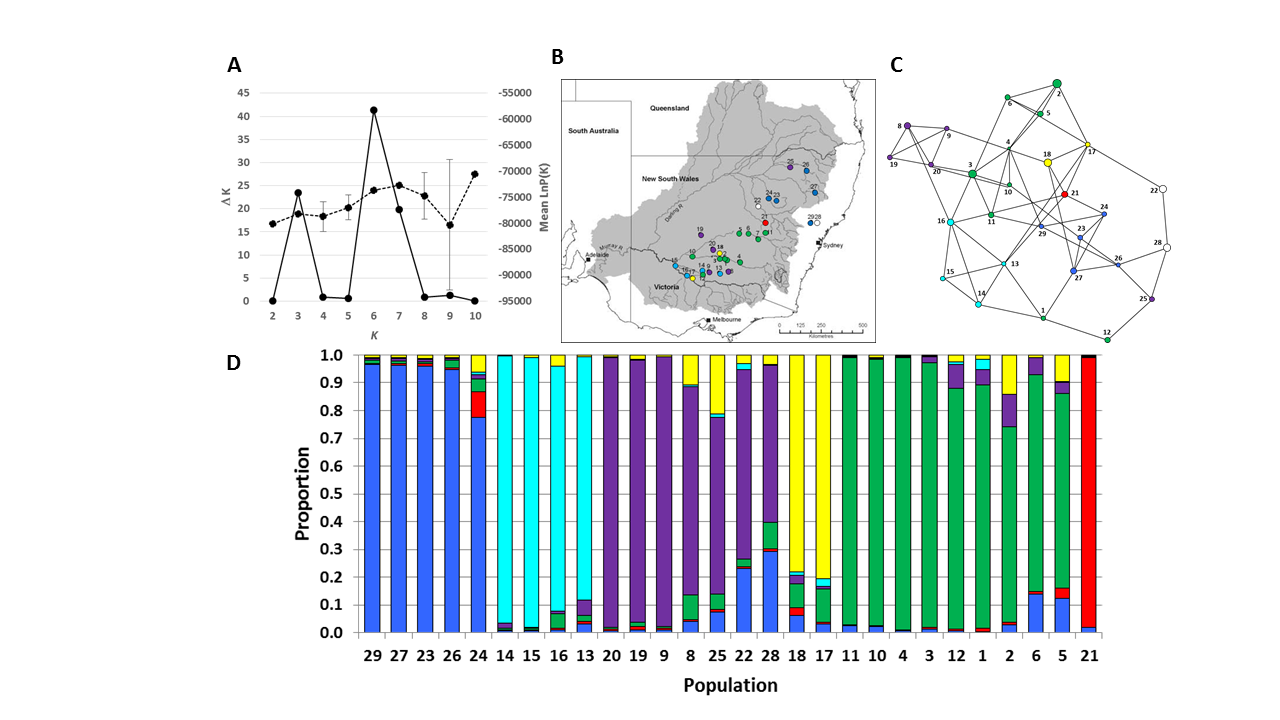


Figure S9. STRUCTURE output and population graph of *A. salicina*. Plot of Δ*K* (solid line) and the mean likelihood (dotted line) for *A. salicina* where the error bar represents one standard deviation (A); geographical location of *A. salicina* sampling locations coloured to match *K*=6, (B); Popgraph plot of *A. salicina* with nodes coloured to match *K*=6 (C); typical *A. salicina* STRUCTURE plot for *K*=6 (D). Sampling location numbers as per Table 1. White nodes indicate <70% assignment to a single cluster.


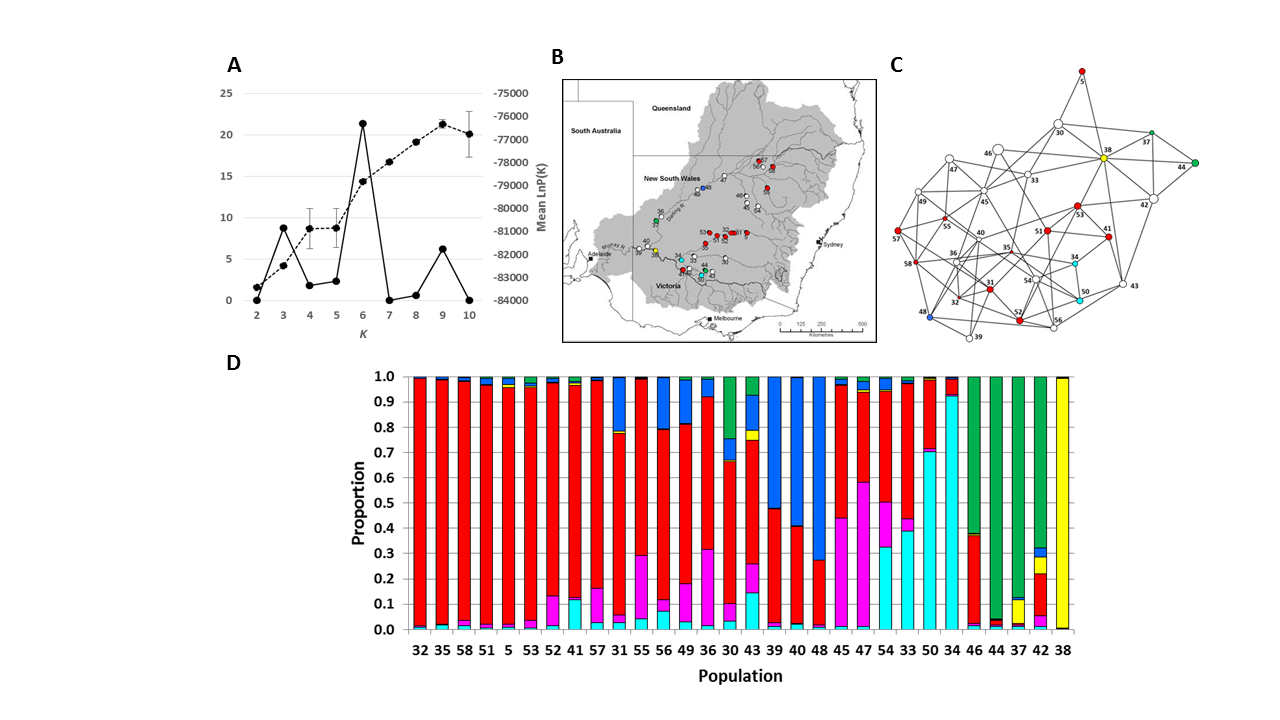


Figure S10. STRUCTURE output and population graph of *A. stenophylla*. Plot of Δ*K* (solid line) and the mean likelihood (dotted line) for *A. stenophylla* where the error bar represents one standard deviation (A); geographical location of *A. stenophylla* sampling locations coloured to match *K*=6, (B); Popgraph plot of *A. stenophylla* with nodes coloured to match *K*=6 (C); typical *A. stenophylla* STRUCTURE plot for *K*=6 (D). Sampling location numbers as per Table 1. White nodes indicate <70% assignment to a single cluster.

**A**

|   **B** |  |
| --- | --- |

Figure S11. STRUCTURE output of *A. salicina* (A) and *A. stenophylla* (B) for K = 3. Geographical location of sampling locations coloured to match K=3. Sampling location numbers as per Table 1. Colours distinguish the genetic clusters inferred from STRUCTURE.

|  |  |
| --- | --- |

Figure S12. Resampling simulation analysis to test sampling size effects in *A. salicina* and *A. stenophylla*. To explore the sample size (i.e. number of individuals) effects of genetic diversity we did a resampling simulation analysis in R. We generated resampled datasets for different number of individuals (number of individuals = 2, 3, 4, 5, 6, 7, and 8 for *A. salicina*, and 2, 3, 4, 5, 6, and 7 for *A. stenophylla*) while keeping fixed the number of replicates (replicates = 1,000). We estimated expected genetic diversity (He) using the function *Hs* from the R package *adegenet* (Jombart 2008). To construct a replicate, individuals were randomly selected without replacement. Sampling location names and numbers can be found in Table S2.

Table S1. Results of MRM for the different resistance surface layers for *A. salicina* and *A. stenophylla.* Significant p values (p < 0.05) are shown in bold*.*

| *Species* | *Model* | *Coefficient values* | *R^2^* | *F* |
| --- | --- | --- | --- | --- |
| 1. *stenophylla* | cGD ~ DEM + LC + FC + WC + GD | Intercept = 21.9 | 0.154 | 15.69 |
|  |  | DEM = -278.4 | - | - |
|  |  | LC = 46.4 | - | - |
|  |  | FC = -22.4 | - | - |
|  |  | WC = 0.002 | - | - |
|  |  | GD = 8.96 e-06 |  |  |
|  | cGD ~ DEM + LC + GD | Intercept = 9.9 | 0.118 | 19.32 |
|  |  | DEM = -2.69 | - | - |
|  |  | LC = 4.31 | - | - |
|  |  | GD = -2.88 | - | - |
|  | cGD ~ DEM + LC | Intercept = 11.94 | 0.117 | 28.85 |
|  |  | DEM = -275.9 | - | - |
|  |  | LC = 39.81 | - | - |
| 1. *salicina* | cGD ~ GD | Intercept = 4.37 | 0.03 | 12.01 |
|  |  | **IBD** = -**2.38e-05** | - | - |

Table S2. Geographical coordinates, names and numbers (IDs) of sampling locations of *A. salicina and A. stenophylla*.

| **Species** | **Sampling location number** | **Latitude** | **Longitude** | **Sampling location name** |
| --- | --- | --- | --- | --- |
| *A. salicina* | 24 | -31.26382 | 148.4589 | Annareb |
| *A. salicina* | 27 | -30.93387 | 150.8973 | Att |
| *A. salicina* | 1 | -34.15135 | 146.3033 | Bernies |
| *A. salicina* | 13 | -35.3596 | 145.7436 | Billsal |
| *A. salicina* | 19 | -33.25785 | 144.7218 | Boxyard |
| *A. salicina* | 18 | -34.27438 | 145.7575 | Bringagee |
| *A. salicina* | 9 | -35.28793 | 145.1749 | Conargo |
| *A. salicina* | 5 | -33.17865 | 146.8193 | Consal |
| *A. salicina* | 2 | -34.61835 | 146.3904 | CubaSF |
| *A. salicina* | 4 | -34.7453 | 146.8152 | GGrong |
| *A. salicina* | 29 | -32.40437 | 150.7293 | Golden |
| *A. salicina* | 15 | -34.9619 | 143.3368 | Goodsal |
| *A. salicina* | 20 | -34.04097 | 145.3893 | Gunbar |
| *A. salicina* | 8 | -35.26862 | 146.2094 | LkUrana |
| *A. salicina* | 6 | -33.21047 | 147.3219 | Lila |
| *A. salicina* | 17 | -35.60637 | 144.2867 | Lowsal |
| *A. salicina* | 3 | -34.67693 | 145.8035 | OppYF |
| *A. salicina* | 11 | -33.14337 | 148.2318 | Parks |
| *A. salicina* | 25 | -29.562 | 149.6196 | Poison |
| *A. salicina* | 12 | -35.70542 | 144.8445 | Pretty |
| *A. salicina* | 16 | -35.47378 | 143.9838 | Sheep |
| *A. salicina* | 21 | -32.57093 | 148.2228 | Toming |
| *A. salicina* | 23 | -31.39413 | 148.8697 | Toor |
| *A. salicina* | 28 | -32.58467 | 151.0307 | Wallaby |
| *A. salicina* | 26 | -29.73335 | 150.5409 | Walliand |
| *A. salicina* | 14 | -35.22825 | 144.8269 | Wansal |
| *A. salicina* | 22 | -31.68448 | 147.835 | Warren |
| *A. salicina* | 10 | -34.47468 | 144.3051 | Yyang |
| *A. stenophylla* | 57 | -29.14617 | 147.48897 | Bangate |
| *A. stenophylla* | 34 | -34.4259 | 143.35253 | Balranald |
| *A. stenophylla* | 56 | -29.3396 | 148.02757 | BigWarr |
| *A. stenophylla* | 43 | -35.19163 | 145.17182 | Billyanco |
| *A. stenophylla* | 47 | -30.3226 | 145.56486 | Bourke |
| *A. stenophylla* | 52 | -33.2369 | 145.59107 | Brewster |
| *A. stenophylla* | 32 | -33.22233 | 146.27031 | Cargellico |
| *A. stenophylla* | 58 | -29.32791 | 148.347 | Collar |
| *A. stenophylla* | 55 | -30.42992 | 148.17297 | Come |
| *A. stenophylla* | 5 | -33.10719 | 147.09559 | Wallamundry |
| *A. stenophylla* | 30 | -34.33512 | 146.01268 | cuba |
| *A. stenophylla* | 50 | -34.31283 | 144.62452 | Hay |
| *A. stenophylla* | 48 | -30.45007 | 144.4657 | Idelia |
| *A. stenophylla* | 37 | -32.69536 | 142.13124 | LkCawndilla |
| *A. stenophylla* | 51 | -33.21188 | 145.33215 | Merrowie |
| *A. stenophylla* | 38 | -34.10588 | 142.10477 | Mildura |
| *A. stenophylla* | 42 | -35.05536 | 144.01473 | Moulamein |
| *A. stenophylla* | 35 | -33.46207 | 144.55228 | Mugga |
| *A. stenophylla* | 46 | -31.10148 | 147.07625 | Murrawombie |
| *A. stenophylla* | 31 | -32.92176 | 146.21576 | MurrinBdg |
| *A. stenophylla* | 33 | -34.284 | 144.17467 | Nap |
| *A. stenophylla* | 39 | -34.03207 | 141.17343 | LkVictoria |
| *A. stenophylla* | 45 | -31.33313 | 147.11098 | Nyngan |
| *A. stenophylla* | 36 | -32.18119 | 142.30065 | Pamamaroo |
| *A. stenophylla* | 40 | -33.55465 | 141.44295 | Tara |
| *A. stenophylla* | 49 | -30.5028 | 144.09291 | Tilpa |
| *A. stenophylla* | 41 | -35.085 | 143.408 | Wakool |
| *A. stenophylla* | 44 | -35.15141 | 144.53345 | Wanganella |
| *A. stenophylla* | 54 | -31.44315 | 147.47005 | WarrenSth |
| *A. stenophylla* | 53 | -33.1144 | 145.07226 | Willandra |

# References

Jombart, T. (2008). adegenet: a R package for the multivariate analysis of genetic markers. *Bioninformatics*, 24, 1403–1405.
